# Popular but precarious: low helmet use among shared micromobility program riders in San Francisco

**DOI:** 10.3389/fpubh.2024.1477473

**Published:** 2024-12-18

**Authors:** Willow Frye, Lara Chehab, Joshua Feler, Laura Wong, Amy Tan, Benjamin Alpers, Devika Patel, Christiana von Hippel, Amanda Sammann

**Affiliations:** ^1^Department of Surgery, University of California, San Francisco, San Francisco, CA, United States; ^2^The Better Lab, University of California, San Francisco, San Francisco, CA, United States

**Keywords:** helmet use behavior, helmet use laws, micromobility, electric bicycle, electric scooter, head injury

## Abstract

**Background:**

Shared micromobility programs (SMPs) are integral to urban transport in US cities, providing sustainable transit options. Increased use has raised safety concerns, notably about helmet usage among e-scooter and e-bicycle riders. Prior studies have shown that head and upper extremity injuries have risen with SMP adoption, yet data on helmet use remains sparse.

**Methods:**

This cross-sectional observational study evaluated helmet use among 5,365 riders (e-bicycles, conventional bicycles, and e-scooters) in San Francisco during February and March 2019. Observations were made at seven key intersections during peak commute hours on clear days.

**Results:**

The majority rode conventional bicycles (77.1%), followed by e-bicycles (19.0%) and e-scooters (3.9%). Most vehicles (82.2%) were personally owned, with the remainder shared via SMPs. Helmet usage was substantially lower among SMP riders, with shared e-scooter users showing the lowest compliance. Specifically, shared e-scooter riders wore helmets 70% less frequently than personal e-scooter riders and 59% less than shared e-bike riders. Dockless e-bike riders used helmets 42% less than those on docked e-bikes.

**Conclusion:**

This study exposes significant gaps in helmet usage among SMP riders, highlighting a pressing need for public health interventions and policy adjustments to improve safety and reduce head injury risks. The findings suggest that helmet use is notably deficient among e-scooter and dockless e-bicycle riders, underscoring the urgent need for targeted safety regulations as cities continue to integrate SMPs into their transportation frameworks.

## 1 Introduction

As urban populations continue to grow and environmental concerns take precedence, cities worldwide are expanding their transportation portfolios to include shared micromobility programs (SMPs). These programs, which encompass shared fleets of e-bicycles, e-scooters, and conventional bicycles, offer a sustainable and flexible transportation alternative (1). However, the rapid adoption and expansion of these services are outpacing the implementation of corresponding safety regulations, particularly around helmet use.

The popularity of SMPs has surged, with the National Association of City Transportation Officials reporting a significant increase in trips over recent years. NACTO’s most recent data reports 130 million shared micromobility trips in 2022, a nearly 400% increase from the 35 million trips reported in 2017 (2). These programs provide inexpensive, convenient and environmentally-friendly options for the consumer, as most riders pay a nominal fee by the minute and can pick up and drop off their vehicle at designated stations across their city (“docked”) or find and leave the vehicle at a location of their choosing (“dockless”). Electric bicycles (“e-bicycles”) and electric scooters (“e-scooters”) also allow the rider to travel longer distance with less effort, making them appealing options for both work and leisure (3). SMPs are coveted by local governments because of their zero emissions, minimal environmental footprint and ability to reduce traffic jams if fewer people drive cars (4, 5).

While these programs reduce carbon footprints and alleviate urban congestion, they also introduce substantial public health challenges, chiefly an increase in traffic-related injuries. Recent studies and reviews of electronic medical records highlight a troubling rise in emergency room visits for injuries related to SMPs, especially head injuries, which are severe yet largely preventable through proper helmet use (5–14). In addition to increasing the volume of riders on the streets at risk for injury, SMPs have increased access to electric vehicles which place riders at risk for more severe injuries. A comparative study in China found that e-bicycle riders are significantly more likely to have traumatic injuries than conventional bicycle riders, and that more than a third of e-bicycle riders had a traumatic brain injury (12). Two studies have found that e-scooter related injuries in the emergency department increased more than 6-fold after the introduction of an SMP in their city (10, 15). A study conducted in Washington, DC, analyzed injuries among riders of electric scooters (e-scooters) and bicyclists, revealing stark differences in helmet usage rates. Less than 2% of injured e-scooter riders were wearing helmets, contrasting sharply with the 66% helmet usage rate among injured bicyclists (16). Furthermore, the study indicated a greater burden of severe injury among e-scooter riders such that they were nearly three times more likely to experience a concussion with subsequent loss of consciousness compared to bicyclists. Another study focusing on oral and facial injuries among e-scooter users and bicyclists also underscored the issue of low helmet use, with injured bicyclists and e-scooter users showing minimal to no helmet use, respectively (17). Three recent studies investigating e-scooter injuries in their local hospital’s emergency department found that along with an increase in traumatic injuries for this cohort, zero riders wore a helmet (7, 10, 18). This trend is concerning given the growing body of evidence suggesting higher injury rates among e-scooter riders compared to cyclists (16). Both the American College of Surgeons (ACS) and the American College of Emergency Physicians (ACEP) name helmet use as a critical prevention tool for head injury on both bicycles and scooters (19, 20).

Over the last decade, several observational studies have found that the prevalence of SMP bicycle riders without a helmet ranges from 80 to 90% versus 10–50% among personal bicycle riders in major US cities including Boston, Washington DC, New York City, and Seattle and have highlighted the importance of legislation and enforcement (21–28). A study in Vancouver, Canada, where helmet use is mandatory for all cyclists, found higher rates of helmet use among both personal (78%) and shared (64%) bike riders compared to cities without such laws, yet there was still a notably reduced prevalence of helmet use when the rider was using a shared bicycle (29). However, the effectiveness of mandatory helmet laws remains debated, with some cities like Seattle recently repealing such laws due to concerns about disproportionate enforcement (30). In a 2018 study in Seattle, the rate of dockless bicycle riders without a helmet was 80%, but this was studied before the city repealed its mandatory helmet law in 2022 (27, 30). A systematic review by Hoye found that mandatory bicycle helmet legislation reduced overall head injury rates among cyclists by 20% and reduced serious head injury rates by 55% (31).

In 2014, the city of San Francisco, California joined the Vision Zero Network to commit to a goal of eliminating traffic fatalities and severe injuries by 2024. Yet that goal has not been achieved to date. According to data from Zuckerberg San Francisco General (ZSFG), San Francisco Police Department (SFPD), and San Francisco Emergency Medical Services Agency (SF EMSA), people biking comprise approximately one-fifth of severe and critical injuries in recent years (32). Similar statistics are not available for e-scooters due to limited data collection to date. However, one report notes that “of 32 e-scooter related injuries reported to SFPD in 2018, 19% were severe, 7% involved wearing a helmet, and 13% were injuries to people walking.” (33) According to self-report data to an e-scooter company, 12% of riders in San Francisco reporting collisions also reported helmet use (34). Understanding the factors influencing helmet use among SMP riders is critical in achieving this goal and ensuring the safe integration of micromobility into San Francisco’s urban landscape.

This study aimed to employ observational methods to assess helmet use among riders of both manual and electric micromobility platforms in comparison to riders of personal vehicles in San Francisco, to understand the factors influencing this behavior, and to provide a data-driven foundation for developing interventions aimed at increasing helmet use and reducing the risk of head injuries. The objectives of this study were to: (1) Assess helmet use rates among riders of personal and shared micromobility vehicles in San Francisco, (2) Compare helmet use between different vehicle types and ownership models, (3) Examine potential differences in helmet use between morning and evening commute times. We hypothesized that: (1) Helmet use would be lower among riders of shared vehicles compared to personal vehicles, (2) E-scooter riders would have the lowest rates of helmet use, (3) Helmet use would be higher during morning commute times compared to evenings.

## 2 Methods

### 2.1 Study design

We conducted a cross-sectional study of e-bicycle, conventional bicycle (“c-bicycle”) and e-scooter riders in the city of San Francisco, California between 2/20/2019 and 3/20/2019. Conventional scooters were not included because they were not available for rent through SMPs. Observations were performed by a team of four researchers who were trained by the lead researcher to systematically collect data.

### 2.2 Research ethics approval

Exempt approval was granted through the Institutional Review Board of our institution (reference #255597). The requirement of informed consent was waived for the portion of the study reported on here as it only involved observation of public behavior.

### 2.3 Patient involvement

This study is one part of a larger initiative taking a human-centered design approach to understanding the factors contributing to injury caused by the e-bike and shared bike industry in San Francisco. Patients with injury related to bicycle or scooter transit were involved in honing the research questions and design for the current study through preliminary qualitative interviews that are being reported on elsewhere.

### 2.4 Observation sites

Seven intersections were purposively sampled for observation during two-hour, peak commute times in the morning (7 a.m. – 9 a.m.) and evening (5 p.m. – 7 p.m.) on days without precipitation. Observation sites were chosen to maximize the number of SMP and personal vehicle riders, and to minimize potential repeated sampling of the same rider in multiple observations. High volume intersections were selected based on publicly available data on bicycle-traffic volume (35). Only intersections at the junction of two officially designated bicycle corridors—defined as roads with bicycle-specific infrastructure such as a shared lane, bike lane, or separated lane—were considered. No single bicycle corridor was represented at more than one observation site to minimize potential duplication of riders. Before observations took place, the research team visited each site to ensure the presence of a safe location with clear sightlines from all four sides of the intersection.

### 2.5 Observation procedures

Each observation was carried out over 2 h by two researchers. With four directions to each intersection (north, south, east, and west), each researcher was responsible for documenting riders approaching from two directions. Researchers were positioned at corners of the intersection with clear sightlines of their assigned directions. Researchers were trained to identify vehicle type, ownership status and distribution model by reviewing representative images of the vehicles, and each practiced live coding for 20 min before performing their first observation. As each rider approached the intersection, appropriate codes were entered into and a standardized data collection form on a tablet to record observations in real-time with a timestamp containing the hour and minute of arrival. Riders walking alongside their vehicles were not included. To minimize potential duplicate observations, researchers were instructed to focus on riders actively crossing the intersection rather than those circling or remaining in the area. Additionally, the selection of distinct commuter corridor sites in different parts of the city further reduced the likelihood of observing the same rider multiple times. Inter-observer reliability was assessed by having pairs of researchers independently code the same intersection for a 30-min period at the beginning of the study. Agreement between observers was high (Cohen’s kappa >0.85) for all key variables (vehicle type, ownership status, and helmet use).

### 2.6 Observational coding

A codebook was generated to characterize the vehicle type, ownership status, distribution model and helmet use of observed riders (Table 1). Because docked and dockless options only existed for shared vehicles, the distribution model was not coded for personal vehicles. Vehicle types and ownership status were identified by distinctive markings and form factors including company logos, batteries, and motors. Distribution model was inferred from the company logos because each SMP company at the time of coding used only one model of distribution. Riders holding a helmet or wearing an unsecured helmet were counted as “no” for helmet use.

**Table 1:** Frequencies of observations*.

Vehicle type	Total observations: N (%)	
ALL	5,365 (100.0%)	
	Personal	Shared
C-bike	4,007 (74.7%)	130 (2.4%)
E-bike	265 (4.9%)	755 (14.1%)
Docked	N/a	595 (11.1%)*
Dockless	N/a	160 (3.0%)*
E-scooter	136 (2.5%)	72 (1.3%)
Total	4,408 (82.2%)	957 (17.8%)

*Percentage represents proportion of total observations.

### 2.7 Data analysis

We calculated descriptive statistics to determine overall prevalence of vehicle types, ownership status, distribution model and vehicle power. We calculated prevalence ratios (PRs) with 95% confidence intervals for helmet use among personal vehicle riders and SMP users, including c-bicycles, e-bicycles (docked and dockless), and e-scooters. Chi-square tests were used to assess for differences in helmet use in the morning versus evening among vehicle type subgroups. Fisher’s exact test was used for one small subgroup (shared scooters). Data were analyzed using R version 4.1.2, and significance was defined as a two-sided *p*-value <0.01.

## 3 Results

**Figure 1:**
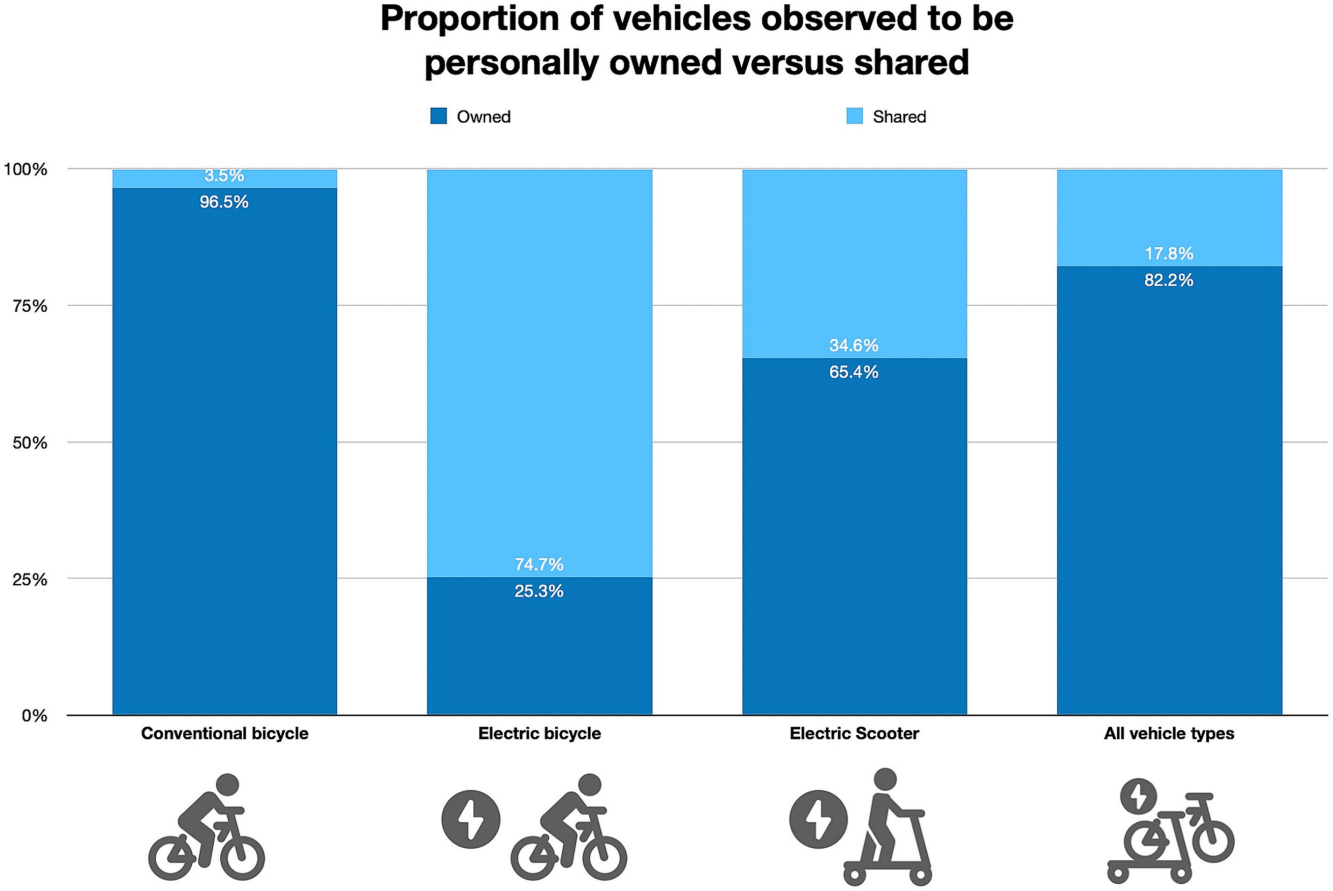
Proportion of vehicles observed to be personally owned versus shared.

A total of 5,365 riders were observed during the study period. Three quarters of rides (77.1%) used c-bicycles (*n* = 4,137), while 19.0% used e-bicycles (*n* = 1,020) and 3.9% used e-scooters (*n* = 208.) A majority (82.2%) of observed vehicles were personal while 17.8% were shared. Regarding bicycle type, personal riders were much more likely to be on a c-bicycle (96.5% vs. 3.5%), whereas, shared riders were more commonly on e-bicycles (74.7% vs. 25.3%) (Figure 1).

**Figure 2:**
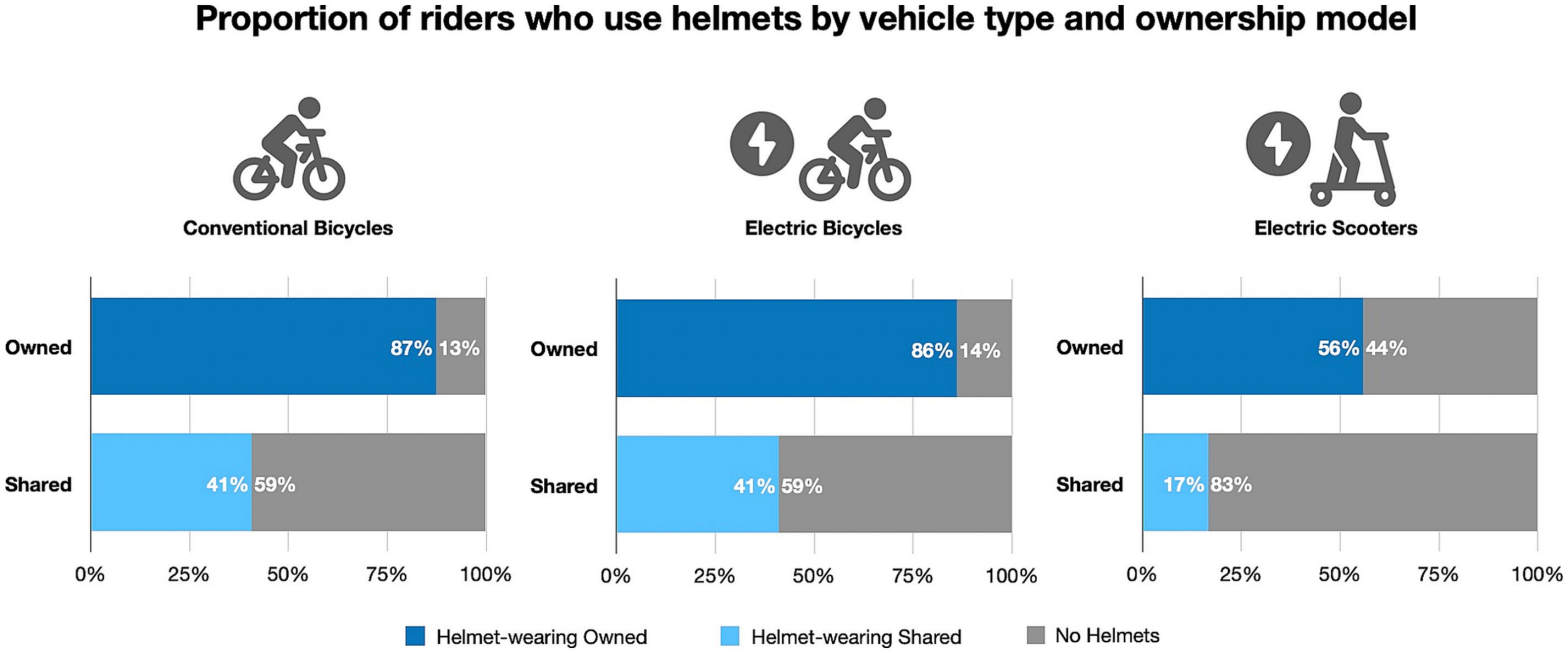
Proportion of riders who use helmets by vehicle type and ownership model.

Riders of shared vehicles wore a helmet less than half of the time than riders of personal vehicles, regardless of vehicle type (Figure 2). There was no significant difference in helmet use between e-bicycles and c-bicycles in riders of personal (PR = 0.99, CI 0.94–1.04, *p* = 0.544) or shared (PR = 0.99, CI 0.79–1.24, *p* = 0.959) vehicles. Riders of shared e-scooters wore a helmet 70% less often than riders of personal e-scooters. Riders of shared e-scooters wore a helmet 59% less often than riders of shared e-bikes. Riders of dockless e-bikes wore a helmet 42% less than riders of docked e-bikes. Riders on personal c-bicycles were significantly more likely to be wearing a helmet in the morning (90%) than in the evening (84%) (Table 2). Otherwise, time of day did not make a significant difference for any other vehicle type (Table 3).

**Table 2:** Primary analysis: prevalence ratios for vehicle types by ownership model.

Vehicle type	Prevalence ratio of helmet use	CI	*P*-value
Personal only			
Personal e-bike vs. Personal c-bike	0.99	0.94–1.04	0.544
Personal e-scooter vs. Personal e-bike	**0.65**	**0.55–0.75**	**<0.0001**
Shared only			
Shared e-bike vs. Shared c-bike	0.99	0.79–1.24	0.959
Shared e-scooter vs. Shared e-bike	**0.41**	**0.24–0.69**	**<0.0001**
Dockless e-bike (shared) vs. Docked e-bike (shared)	**0.58**	**0.44–0.76**	**<0.0001**
Shared vs. Personal			
Shared vs. Personal (All)	**0.45**	**0.41–0.49**	**<0.0001**
Shared c-bike vs. Personal c-bike	**0.47**	**0.38–0.57**	**<0.0001**
Shared e-bike vs. Personal e-bike	**0.47**	**0.43–0.52**	**<0.0001**
Shared e-scooter vs. Personal e-scooter	**0.30**	**0.17–0.51**	**<0.0001**

Bold values indicate statistical significance of the result.

**Table 3:** Subgroup analysis: morning vs. evening rider helmet use by vehicle type.

Vehicle type	Personal			Shared		
	Morning	Evening % (n)	p-value	Morning	Evening	*p*-value
C-bike	90% (1974/2195)	84% (1,525/1812)	<0.001	40% (36/90)	43% (17/40)	0.94
E-bike	85% (100/117)	86% (128/148)	0.95	42% (216/508)	36% (90/247)	0.13
*Docked*	N/a	N/a	N/a	45% (224/497)	41% (94/228)	0.37
*Dockless*	N/a	N/a	N/a	25% (36/146)	20% (17/86)	0.49
E-scooter	55% (60/110)	62% (16/26)	0.67	18% (8/45)	15% (4/27)	>0.99
All	**88% (2,134/2422)**	**84% (1,669/1986)**	**<0.001**	40% (260/643)	35% (111/314)	0.15

Bold values indicate statistical significance of the result.

## 4 Discussion

This study demonstrated that SMP riders are significantly less likely to wear a helmet compared to riders of personal vehicles, supporting our first hypothesis. Our second hypothesis was also supported, as e-scooter riders, particularly those using shared e-scooters, had the lowest rates of helmet use. However, our third hypothesis was only partially supported; while we observed higher helmet use rates in the morning for personal c-bicycle riders, this pattern did not hold true for other vehicle types. The observed helmeted rates of c-bicycles (41% shared, 87% personal) and e-bicycles (41% shared, 86% personal) were higher than rates found in previous studies in different cities (17, 18, 20, 23, 24). However, the observe helmeted rates for shared dockless bikes (26%) and shared e-scooters (17%) were much lower than the other types, aligning with findings from Hoye’s systematic review (31).

Given the growing popularity of SMPs and electric vehicles and a growing body of evidence documenting an increase in traumatic injury associated with their use, these groups represent important cohorts for targeted public health and safety interventions. Of note, Ioannides, et al. found the rate of injury among e-scooter riders in Los Angeles to be 115 per million trips, 7.7 times the injury rate among bicycle riders and 14.3 times the injury rate of passenger car trips (36). Among 249 encounters for standing electric scooter injuries at 2 urban emergency departments associated with an academic medical center in Southern California in 2018, 40.2% involved a head injury, of which 5% resulted in intracranial hemorrhage (7).

While the effects of helmet use for reducing risk for serious head injury is well-documented and accepted by public health authorities, there is a lack of consistent helmet legislation and enforcement in the US (4, 31). Globally, approaches to regulating shared micromobility and helmet use vary widely. For instance, Buongiorno et al. conducted a comparative analysis of e-scooter regulations across European countries, finding significant variation in helmet requirements and enforcement strategies (37). The same study also proposed increased efforts to ensure the availability of helmets at SMP stations and the implementation of safety enhancements to the e-scooters themselves including the addition of rearview mirrors, horns, and turn signals accessible on the handlebars. Interestingly in Vancouver, where at the time of the study, there was both a mandatory helmet law for all vehicles including SMPs, the helmeted rates among shared (64%) and personal (79%) riders were much higher than other cities. However, cities like Seattle have recently repealed their mandatory helmet laws due concerns of minimal enforcement that was disproportionately used against Black and unhoused riders (30). In New South Wales, Australia, we can observe long-term success where a combination of helmet legislation, enforcement, and public education campaigns has led to sustained increases in helmet use and reductions in head injuries among cyclists over the decades since legislation was first implemented in 1991 (38). Indeed communication campaigns have been shown to serve as a valuable complement to other legislative and educational approaches to reducing road injury prevalence and severity (39). As San Francisco and other cities work toward a goal of eliminating traffic fatalities and severe injuries as laid out by the Vision Zero Network, serious consideration should be given to policies and programs that have worked to increase helmet use, especially among SMP users, in other countries.

Future research must broaden our understanding on helmet use to include other safety behaviors such as: alcohol use, adherence to stop signs and red lights, riding against traffic flow, riding on sidewalks, riding one handed, wearing headphones, and riding with a passenger. Specific concerns include intoxicated riding due to the availability of SMPs in nightlife areas, increased use by very young or very old riders, and differences in how riders navigate the environment that might induce more crashes or cause more severe injuries, such as head on collisions (7). Research should include trip purpose and timing, and rider demographics, as well as the built environment and corresponding automobile driver safety behaviors. It is imperative that we understand factors driving helmet use, or the lack of it, among riders of shared and electric vehicles to design interventions that can lead to meaningful injury interventions.

### 4.1 Study limitations

Our study had several limitations. Due to the observational nature of this study, sample sizes for the vehicle type and power are unbalanced - leading to sparsity in subgroups and reduced generalizability of the results. Convenience sampling within this study design also prevented us from collecting rider characteristics such as age, gender or purpose of the ride, which may be modifying factors contributing to helmet use. More data are therefore required to balance the subgroups, increase generalizability and understand additional individual characteristics as they relate to helmet use. Second, it is possible that the same rider may have appeared in multiple observations despite the selection of distinct commuter corridor sites in an effort to mitigate this possibility. Third, our data do not account for helmet use during inclement weather because precipitation was a study exclusion criterion. Because our study took place on clear days from February 20th 2019 – March 20th 2019, seasonal variation was not assessed. Additionally, our focus on commute hours may not capture helmet use patterns during other times of day or for non-commute trips, potentially limiting the generalizability of our findings to overall micromobility use in San Francisco.

## 5 Conclusion

This investigation highlights a pressing public health issue—low helmet use in SMP riders in San Francisco-and highlights that SMP riders are significantly less likely to wear a helmet compared to riders of personal vehicles. Our results call for immediate attention from local policymakers to enforce helmet use among SMP users through new evidence-based policies and develop educational programs aimed at reducing head injury risks in urban micromobility settings.

## Data Availability

The raw data supporting the conclusions of this article will be made available by the authors, without undue reservation.
